# Polysaccharides from Spores of *Cordyceps cicadae* Protect against Cyclophosphamide-Induced Immunosuppression and Oxidative Stress in Mice

**DOI:** 10.3390/foods11040515

**Published:** 2022-02-11

**Authors:** Yi Zheng, Shiying Li, Chuang Li, Ying Shao, Anhui Chen

**Affiliations:** School of Food and Biological Engineering, Xuzhou University of Technology, Xuzhou 221018, China; lsy842426@163.com (S.L.); a1484262436@163.com (C.L.); shyzhbo2005@126.com (Y.S.)

**Keywords:** *Cordyceps cicadae*, spore, polysaccharide, immunomodulatory, cyclophosphamide

## Abstract

This study investigated the purification, preliminary structure and in vivo immunomodulatory activities of polysaccharides from the spores of *Cordyceps cicadae* (CCSP). The crude CCSP was purified by diethylaminoethyl (DEAE)-cellulose and Sephadex G-100 chromatography, affording CCSP-1, CCSP-2 and CCSP-3 with molecular weights of 1.79 × 10^6^, 5.74 × 10^4^ and 7.93 × 10^3^ Da, respectively. CCSP-2 consisted of mannose and glucose, while CCSP-1 and CCSP-3 are composed of three and four monosaccharides with different molar ratios, respectively. CCSP-2 exhibited its ameliorative effects in cyclophosphamide-induced immunosuppressed mice through significantly increasing spleen and thymus indices, enhancing macrophage phagocytic activity, stimulating splenocyte proliferation, improving natural killer (NK) cytotoxicity, improving bone marrow suppression, regulating the secretion of cytokines and immunoglobulins, and modulating antioxidant enzyme system. These results indicate that CCSP-2 might be exploited as a promising natural immunomodulator.

## 1. Introduction

*Cordyceps cicadae*, an entomogenous fungus used as food and medicine, belongs to the Clavicipitaceae family of the order Hypocreales, which is formed by cicada nymphs infected with *Isaria cicadae* (*Paecilomyces cicadae*), and its morphological structure is composed of spores, sclerotium and coremium (fruiting bodies). It is one of the earliest recorded and well-known traditional Chinese medicine for treating convulsions, asthma, measles, insomnia, chronic kidney diseases and heart palpitations [1]. Modern pharmacological studies have shown that *C. cicadae* possesses an extensive range of pharmacological effects including antioxidant [2], immunomodulatory [3], antitumor [4], renal protective [5], neuroprotective [6], antibacterial [7], hepatoprotective [8] and anti-diabetic [9] activities.

Polysaccharides are the best-known bioactive components derived from *C. cicadae*. Recently, there has been an increasing number of investigations into polysaccharides from *C. cicadae*. Olatunji et al. [10] demonstrated that two *C. cicadae* polysaccharides (namely CPA-1 and CPB-2) can protect PC12 cells against glutamate-induced oxidative toxicity. Zhang et al. [7] found that a *C. cicadae* polysaccharide exerted its antibacterial activity through damaging cell walls and membranes, increasing the permeability of cell membranes. Xu et al. [11] compared the pharmacological activity of two polysaccharides from *C. cicadae* (JCH-1 and JCH-2), and found that the immunomodulatory activity of JCH-1 with low molecular weight was more potent than that of JCH-2 with high molecular weight. In a follow-up study, it was demonstrated that JCH-1 could active RAW264.7 cells via toll-like receptor 4 (TLR4) mediated mitogenactivated protein kinase (MAPK) and nuclear factor-kappa B (NF-κB) signaling pathway [12]. A recent paper demonstrated that polysaccharides from the mycelium and coremium of *C. cicadae* exhibited hypoglycemic, hypolipidemic and antioxidant effects in diabetes rats [13]. Yang et al. [14] found that a nondigestible polysaccharide from the fruiting bodies of artificially cultivated *C. cicadae* remarkably inhibited nitric oxide, interleukin 1β (IL-1β) and tumor necrosis factor-alpha (TNF-α) levels in lipopolysaccharide (LPS) induced RAW264.7 macrophages. Zhu et al. [2] found that CP70, a polysaccharide from *C. cicadae* prepared by the final ethanol concentration of 40%, could extend the lifespan of Drosophila by up-regulating antioxidant enzyme gene expression, thus it demonstrates strong antioxidant and anti-aging activities.

Previously published studies have tended to focus on polysaccharides from the coremium and mycelium rather than spores of *C. cicadae*. The spores are gradually transformed from the active components of the sclerotium and coremium. They have a reproductive function and contain all the genetic materials and biologically active ingredients of *C. cicadae*. It is believed that the pharmaceutical values of the spores could be higher than those of the mycelium and coremium. Sun et al. [15] demonstrated that *C. cicadae* spore powders exerted antiproliferative activities on A549 lung cancer cells by the epithelial mesenchymal transition, Wnt/β-catenin signal pathway and mitochondrial apoptotic pathway. Recently, numerous bioactive substances from the spores of *C. cicadae* were identified and characterized, such as cordycepic acid, nucleosides, cordycepin, beauvericin, sterols and cyclodesipeptide [15,16]. However, up to now, there have been few reports on polysaccharides from spores of *C. cicadae* (CCSP), involving the difficulties associated with the collection.

Recently, researchers have shown an increased interest in polysaccharides from spores of *Ganoderma lucidum* (GLSP) for their versatile pharmacological activities, such as immunostimulatory, antioxidant and antitumor activities [17,18,19,20]. GLSP injection (GuoYaoZhunZi H20003510 and H20003123) has been approved by State Drugs Administration of China for the treatment of neurosis, polymyositis, dermatomyositis and progressive muscular dystrophy, as well as various diseases caused by a weakened immune system. Furthermore, Lentinan, a β-(1→3)-d-glucan derived from *Lentinus edodes*, is one of the best-known bioactive polysaccharides, which exerts antitumor activity by regulating the immune function [21]. Lentinan injection (GuoYaoZhunZi Z10920012 and H20030131) and Lentinan capsule (GuoYaoZhunZi Z20080579) have been approved as an immunomodulator for the adjuvant therapy of cancers in China. Clinical studies have shown that the combination of Lentinan and conventional chemotherapy drug exerts a synergistic effect in prolonging the survival time and improving the quality of life during cancer treatment [22]. These facts prompted us to investigate whether CCSP have immunomodulatory activities.

Cyclophosphamide (CTX) is a broad-spectrum alkylating agent for cancer treatment, which has curative effects on leukemia and solid tumors, and is also one of the most commonly used immunosuppressants [23]. In mammalian liver, CTX is catalyzed by cytochrome P450 enzymes into phosphoramide mustard and acrolein. The former exerts antitumor effects, while the latter can cause severe adverse effects such as immunosuppression, oxidative stress and gut microbiota imbalance [24,25,26]. Studies have found that high-dose or long-term low-dose CTX can induce host immune suppression. A considerable number of studies have evidenced that natural polysaccharides could alleviate CTX-induced immunosuppression and oxidative stress [26,27,28,29].

In the current study, we isolated and characterized polysaccharides from the spores of *C. cicadae*, investigated in vivo immunostimulatory activity in CTX-induced immunosuppressed mice, and endeavored to elucidate the underlying mechanism.

## 2. Materials and Methods

### 2.1. Materials and Chemicals

DEAE-cellulose (DE-52), Sephadex G-100, dextran standards (1, 12, 50, 80, 270, 670 and 1100 kDa) and monosaccharide standards were purchased from Sigma (St. Louis, MO, USA). CTX was acquired from Shanxi Powerdone Pharmaceutics Co., Ltd. (Datong, China). Lentinan (GuoYaoZhunZi Z20080579) was supplied by Hubei Chuangli Pharmaceutical Co., Ltd. (Zaoyang, China). RPMI-1640 medium was acquired from Gibco (Grand Island, NE, USA). Giemsa stain was supplied by Shanghai Yeasen Biotechnology Co., Ltd. (Shanghai, China). TNF-α, IL-1β, Immunoglobulin A (IgA), immunoglobulin G (IgG) and immunoglobulin M (IgM) kits were acquired from R&D system (Minneapolis, MN, USA). Catalase (CAT), glutathioneperoxidase (GSH-Px), superoxidase dismutase (SOD), malondialdehyde (MDA) and protein carbonyl group (PCG) kits were supplied by Jiancheng Bio-engineering Institute (Nanjing, China).

### 2.2. Preparation of Polysaccharides

*I. cicadae* was growth in liquid medium (1% complex amino acids, 1% soy protein hydrolysate, 1% yeast extract, 3% sucrose and 1% glucose) at 25 °C for 7–10 d, then the seed culture was transferred into solid medium (80% wheat bran, 15% buckwheat flour, 2% corn flour, 2% silkworm chrysalis powder and 1% yeast extract). After incubation at 18 °C for 10 d, the solid medium was transferred to a light incubator at 21 °C for 21–28 d, and the spores were collected. 500 g spores of *C. cicadae* were firstly degreased with petroleum ether and pretreated with 95% ethanol twice. Then the precipitates were collected by centrifugation (3500 r/min for 10 min) and dried to constant weight. Enzyme-assisted extraction of CCSP was carried out as follows: ratio of water to raw material 35 mL/g, cellulase amount 0.64%, citrate buffer solution pH 6.0, hydrolysis time 80 min and hydrolysis temperature 50 °C. After extraction, the enzyme was inactivated at 100 °C for 10 min. The extracting solution was filtered, concentrated, precipitated with 70% ethanol at 4 °C for 12 h, and centrifuged at 3000 r/min for 10 min. Then the precipitate was collected and deproteinized with Sevag reagent. The deproteinized solution was dialyzed (molecular weight cut-off: 4000 Da) for 48 h, concentrated and freeze-dried to obtain crude CCSP. The polysaccharide content was measured using the phenol-sulfuric acid method [30].

The crude CCSP were purified by DE-52 cellulose and Sephadex G-100 chromatography according to the previous method [31]. Briefly, 5 mL of polysaccharides solution (20 g/L) were sampled to a DE-52 cellulose column (2.6 cm × 30 cm), and stepwisely eluted with different concentrations of sodium chloride (0, 0.1, 0.3, and 0.5 mol/L), and the elution flow rate is 1 mL/min. As a result, three fractions were obtained, concentrated, dialyzed, and further purified by Sephadex G-100 column (2.6 cm × 60 cm) eluted with deionized water at the flow rate of 0.5 mL/min to afford CCSP-1, CCSP-2 and CCSP-3, respectively. Then they were freeze-dried for further analysis.

### 2.3. Characterization of Polysaccharides

#### 2.3.1. Carbohydrate, Protein and Uronic Acid Analysis

Carbohydrate, protein and uronic acid content of crude CCSP, CCSP-1, CCSP-2 and CCSP-3 were determined using the phenol-sulfuric acid method, Bradford method [32] and m-hydroxydiphenyl method [33], respectively.

#### 2.3.2. Determination of Molecular Weight

High performance gel permeation chromatography (HPGPC) was employed to determine the molecular weights of CCSP-1, CCSP-2 and CCSP-3. The HPGPC conditions were as follows: Agilent 1200 HPLC system (Agilent Technologies, Santa Clara, CA, USA), TSKgel^®^ G5000PW_XL_ gel chromatography column (7.8 mm × 300 mm, Tosoh, Tokyo, Japan), column temperature of 28 °C, 0.002 mol/L sodium dihydrogen phosphate (containing 0.05% NaN_3_) employed as mobile phase, flow rate of 0.6 mL/min, G1352A refractive index detector (Agilent Technologies). Dextran standard solution (2 mg/mL, 20 μL) with molecular weights of 1000, 5000, 12,000, 50,000, 270,000 and 1,100,000, respectively, was injected into the column in order of increasing molecular weight. The retention time was recorded, and the standard curve was plotted. Under the same detection conditions, the retention times of the polysaccharide solutions (1 mg/mL) were recorded, and the molecular weights of the polysaccharides were calculated according to the standard curve.

#### 2.3.3. Monosaccharide Composition

The monosaccharide composition and molar ratio of crude CCSP and its purified fractions were determined by gas chromatography-mass spectrometry (CG-MS). 5 mg polysaccharide samples were dissolved in 5 mL 2 mol/L trifluoroacetic acid (TFA), hydrolyzed at 99 °C for 5 h, then TFA was removed by rotary evaporation. 0.5 mL 4% sodium borohydride was added, placed at room temperature for 1.5 h. Acetic acid was added to remove the sodium borohydride, and the unreacted acetic acid was further removed by rotary evaporation. After vacuum drying, pyridine (1 mL) and n-propylamine (1 mL) were added to the above reaction system, incubated at 55 °C for 30 min, and further dried in vacuum. In total, 0.5 mL each of pyridine and acetic anhydride was added in a water bath at 95 °C for 1 h. The products were blow-dried by nitrogen, dissolved by chloroform, and analyzed by GC-MS.

The GC-MS parameters were as follows: Agilent 7890A/5975C (Agilent Technologies, Santa Clara, CA, USA), DB-5 column (30 m × 0.25 mm × 0.25 μm), mass detector, inlet temperature of 250 °C, detector temperature of 280 °C, helium flow rate of 0.6 mL/min, split ratio of 20:1, injection volume of 5 μL. The column temperature was set at 200 °C for 2 min, increased to 245 °C at 3 °C/min and to 270 °C at 10 °C/min for 2 min.

#### 2.3.4. Infrared Spectral Analysis

Fourier transform infrared (FT-IR) spectra of crude CCSP and CCSP-1, CCSP-2 and CCSP-3 were detected by a Nicolet iS5 spectrometer (Thermo Fisher Scientific Inc., Waltham, MA, USA) with the wavenumber ranged from 4000 to −500 cm^−1^.

#### 2.3.5. Scanning Electron Microscope (SEM) Analysis

CCSP-1, CCSP-2 and CCSP-3 were glued to the sample table with a conductive glue, coated with a thin layer of gold, and the morphology was observed with a Sigma 500 field emission SEM (Carl Zeiss, Oberkochen, BW, Germany).

### 2.4. Immunomodulatory Activities of CCSP-2 in the CTX-Induced Immunosuppressed Mice

#### 2.4.1. Animal Grouping and Experimental Design

C57BL/6 mice (18–22 g) were supplied by Jinan Pengyue Laboratory Animal Breeding Co., Ltd. (production license No. SCXY (Lu) 20190003, Jinan, China). All mice were acclimatized for a week at 22 ± 2 °C with 45–60% relative humidity. All experiments were approved by the Institutional Animal Care and Use Committee.

The mice were randomly divided into six groups (*n* = 10): normal group, model group, positive control group and three CCSP-2 treated groups. Each group was half male and half female. The mice were intraperitoneally injected with 120 mg/kg CTX once a day for three consecutive days, except for the normal group. Then the low-, medium- and high-dose CCSP-2 groups were intragastrically administered with 50, 100 and 200 mg/kg CCSP-2, respectively, once a day for 21 consecutive days. The doses of CCSP-2 selected were based on pre-experiment and previous studies [34,35,36]. The positive control group mice were intragastrically with 150 mg/kg Lentinan, the dose selected was based on an estimated human-equivalent dose; the normal group and model group mice were given normal saline.

The mice were weighed and sacrificed by cervical dislocation after 24 h the last gavage, then the thymus and spleen were removed, and the organ index was measured as the ratio of organ mass to body weight.

#### 2.4.2. Peripheral Blood Counting Assay

Blood samples were collected from the retro-orbital venous plexus into heparin tubes. The number of white blood cells, neutrophils, lymphocytes and platelets were counted by a SYsmex XT-2000iV blood analyzer (Sysmex Partec, Kobe, Japan).

#### 2.4.3. Macrophage Phagocytosis Assay

The mice were intraperitoneally injected with 2 mL of 1% chicken red blood cells (CRBCs) suspension after 24 h the last treatment, and the chicken cells were dispersed by gently rubbing the abdomen. After 30 min, the mice were sacrificed, intraperitoneally injected with 2 mL phosphate buffer solution, then the peritoneal fluid was collected by gently rubbing the abdomen. The peritoneal fluid was deposited onto a glass slide and incubated for 30 min at 37 °C. The cells that were not adhered to the slide were removed with water, then the slide was fixed with acetone-methanol solution, stained with Giemsa, rinsed with water and dried. The peritoneal macrophages were counted, and the phagocytosis rate was measured as follows:

Phagocytic rate (%) = (number of macrophage-ingesting CRBCs/total number of macrophages) × 100(1)

#### 2.4.4. Splenic Lymphocyte Proliferation Assay

The spleens of each group mice were aseptically removed, added to pre-cooled Hank’s solution, gently ground, filtered through a 200-mesh sieve, washed with Hank’s solution twice, centrifuged at 1500 r/min for 5 min, and the splenic lymphocytes were collected. The cell concentration was adjusted to 3.0 × 10^6^ cells/mL and inoculated in a sterile plate with 100 μL per well. Concanavalin A (Con A) solution (final concentration of 7.5 μg/mL) or LPS solution (final concentration of 10 μg/mL) was added into each well. It was incubated in 5% CO_2_ and 37 °C incubator for 72 h. Then 20 μL MTT solution (5 mg/mL) was added and incubated for 4 h. The cell plate was taken out, the supernatant was discarded, 150 μL dimethyl sulfoxide (DMSO) was added into each well, and a microplate shaker was used to dissolve the crystals. The absorbance at 570 nm was detected by a Biotek Synergy2 multi-mode plate reader.

#### 2.4.5. NK Cytotoxicity

The effect of CCSP-2 on NK cytotoxicity in CTX-induced immunosuppressed mice was determined, with spleen lymphocytes as effector cells and YAC-1 cells as target cells. The splenic lymphocytes were collected as described above, and added into the 96-well plate with 5 × 10^5^ cells per well. The target cells were added at a ratio of target to effector cells of 1:50, and incubated in 5% CO_2_ at 37 °C for 10 h. 5 mg/mL MTT (50 μL) was added into each well and incubated for 4 h. Then 100 L DMSO was added, mixed with a microplate shaker, and the absorbance at 570 nm was detected. The NK cytotoxicity was measured as follows:

NK cytotoxicity (%) = [*A*_t_ − (*A*_s_ − *A*_e_)]/*A*_t_ × 100 (2)

where *A*_t_, *A*_s_, and *A*_e_ are, respectively, the absorbances of target cells control, sample, and effector cells control.

#### 2.4.6. Serum Cytokine and Immunoglobulin Assay

Blood samples were obtained from the retro-orbital venous plexus, centrifuged at 3000 r/min for 15 min at 4 °C, and the serum was obtained. The levels of IL-1β, TNF-α, IgA, IgG and IgM were determined according to the kit instructions.

#### 2.4.7. Antioxidase and Oxidative Damage Product Assay

The liver tissues were removed, cut into pieces and added to pre-cooled normal saline, homogenized in ice-cold buffer, centrifuged at 3500 r/min at 4 °C for 15 min, then the supernatant was collected. SOD, CAT, GSH-Px activities and MDA, PCG levels in the liver tissues were measured in accordance with the kit instructions.

### 2.5. Statistical Analysis

The results are expressed as mean ± SD. The statistical significance between test groups and control group was analyzed by one-way analysis of variance (ANOVA) with Dunnett-*t* test. *p* < 0.01 and *p* < 0.05 denote highly significant difference and significant difference, respectively.

## 3. Results

### 3.1. Purification of CCSP

**Figure 1:**
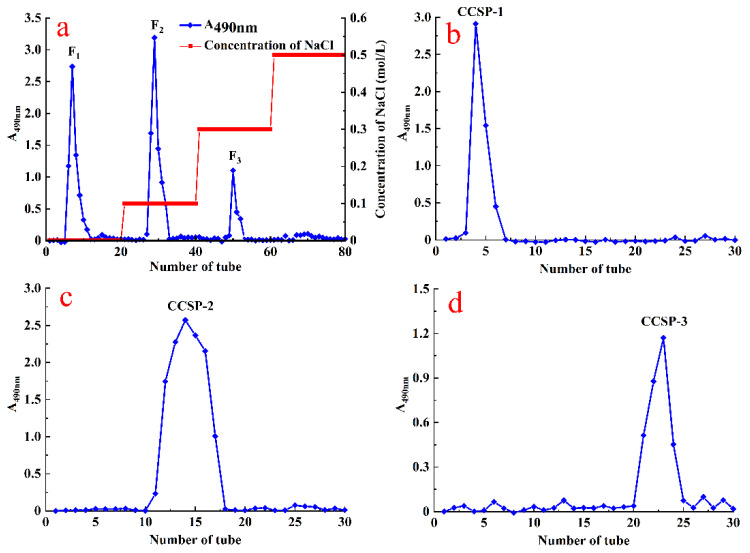
Stepwise elution curve of crude polysaccharides from spores of *C. cicadae* (CCSP) on DE-52 cellulose chromatography (**a**) and elution curve of F_1_, F_2_ and F_3_ on Sephadex G-100 chromatography affording CCSP-1 (**b**), CCSP-2 (**c**) and CCSP-3 (**d**).

Crude CCSP were firstly separated by DEAE-cellulose chromatography resulting in three peaks (F_1_, F_2_ and F_3_) (Figure 1a), which were collected, concentrated, dialyzed and loaded into the Sephadex G-100 column affording CCSP-1, CCSP-2 and CCSP-3, respectively (Figure 1b–d). Moreover, the recovery rates of CCSP-1, CCSP-2 and CCSP-3 based on the amount of crude CCSP were 29.42%, 35.26% and 5.43%, respectively.

### 3.2. Characterization of CCSP and Its Purified Fractions

#### 3.2.1. Contents of Carbohydrate, Protein and Uronic Acid

The carbohydrate, protein and uronic acid contents of crude CCSP and its purified fractions are displayed in Table 1. The carbohydrate contents of crude CCSP, CCSP-1, CCSP-2 and CCSP-3 were 74.15 ± 3.23%, 97.94 ± 4.84%, 96.65 ± 5.32% and 90.43 ± 4.60%, respectively. The protein content of CCSP-3 was 3.18 ± 0.05%, and no protein was detected in CCSP-1 and CCSP-2, suggesting that CCSP-3 could be a protein-polysaccharide complex. In addition, CCSP-1 should be a neutral polysaccharide, while CCSP-2 and CCSP-3 should be acidic polysaccharides.

#### 3.2.2. Molecular Weight Distribution

**Figure 2:**
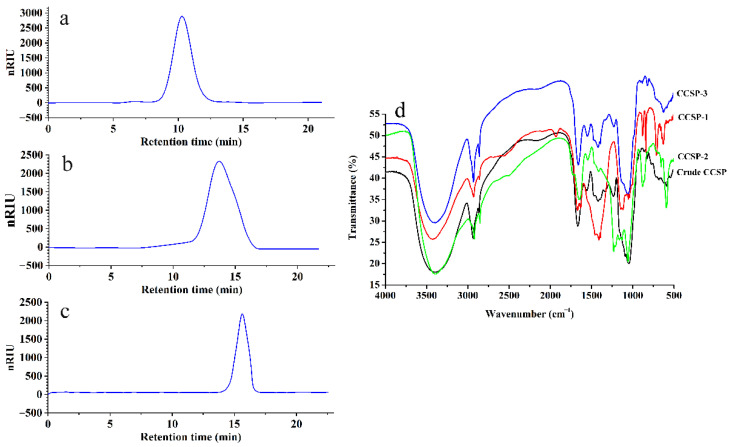
High performance gel permeation chromatography (HPGPC) profiles of CCSP-1 (**a**), CCSP-2 (**b**) and CCSP-3 (**c**) and Fourier transform infrared (FT-IR) spectra of crude CCSP and its purified fractions (**d**). The HPGPC conditions were as follows: Agilent 1200 HPLC system, TSKgel^®^ G5000PW_XL_ gel chromatography column, column temperature of 28 °C, 0.002 mol/L sodium dihydrogen phosphate (flow rate of 0.6 mL/min) used as mobile phase, G1352A refractive index detector. FT-IR spectra were determined by a Nicolet iS5 spectrometer with the wavenumber ranged from 4000 to cm^−1^.

HPGPC was employed to determine the molecular weights of the purified polysaccharide fractions, as shown in Figure 2a–c. CCSP-1, CCSP-2 and CCSP-3 all showed a single sharp and symmetrical peak, suggesting that they were homogeneous polysaccharide fractions. The standard curve equation of dextran obtained by HPGPC was *y* = −0.4429*x* + 10.827, *R*^2^ = 0.9925, where *x* was the retention time on the chromatographic column, and *y* was the logarithm of the molecular weight. According to the retention time, the molecular weights of CCSP-1, CCSP-2 and CCSP-3 were calculated as 1.79 × 10^6^, 5.74 × 10^4^ and 7.93 × 10^3^ Da, respectively.

#### 3.2.3. Monosaccharide Composition

According to GC-MS analysis, glucose was found to be the main monosaccharide in crude CCSP and its purified fractions. The crude CCSP consisted of xylose, mannose, glucose and galactose. CCSP-1 was composed of mannose, glucose and galactose, and CCSP-2 consisted of only glucose and mannose with a molar ratio of 94.27:5.73. CCSP-3 was composed of xylose, mannose, glucose and galactose with a molar ratio of 22.08:2.05:63.40:12.27, suggesting that the monosaccharide composition of CCSP-3 was more complicated than those of CCSP-1 and CCSP-2.

#### 3.2.4. Infrared Spectral Analysis

The FT-IR spectra of crude CCSP, CCSP-1, CCSP-2 and CCSP-3 are illustrated in Figure 2d. The strong and wide absorption peak ranged from 3370 to 3410 cm^−1^ were related to O-H stretching vibration, and the absorption peaks at 2918–2930 cm^−1^ were reasonably assigned to C-H stretching vibration. These two groups’ peaks are the characteristic absorptions of polysaccharides. Absorption peaks at 1646–1656 cm^−1^ and 1400–1420 cm^−1^ were ascribed to the asymmetric and symmetric stretching vibrations of C=O, respectively, indicating that the polysaccharide samples contained carboxyl groups, which was consistent with the results of the above chemical analysis. Moreover, stretching vibration of S=O and C-O-C could account for the absorption peaks at 1220–1245 cm^−1^ and 1049–1077 cm^−1^, respectively. The absorption peaks at 810 and 870 cm^−1^ revealed the existence of a mannose residue in the polysaccharide samples.

#### 3.2.5. Morphology Analysis

**Figure 3:**
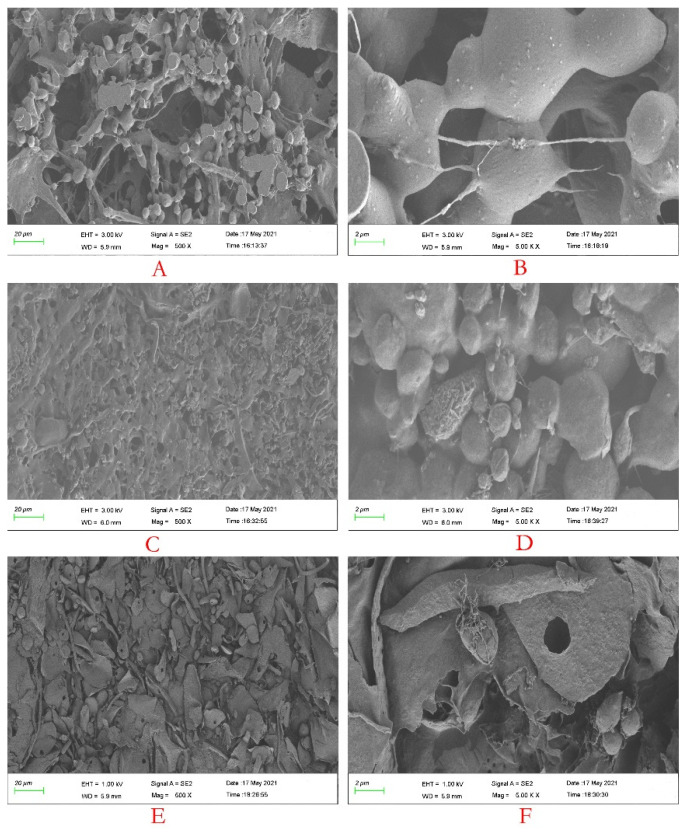
Scanning electron microscope(SEM) images of CCSP-1 (**A**,**B**), CCSP-2 (**C**,**D**) and CCSP-3 (**E**,**F**). (**A**,**C**,**E**) 500×; (**B**,**D**,**F**) 5000×. CCSP-1, CCSP-2 and CCSP-3 were glued to the sample table with a conductive glue, coated with a gold layer, and the morphology was observed with a Sigma 500 field emission SEM.

Figure 3 shows the SEM images of CCSP-1, CCSP-2 and CCSP-3. When the magnification was 500×, CCSP-1 (A), CCSP-2 (C) and CCSP-3 (E) showed loose granular and filamentous accumulations, dense granular and filamentous aggregates, and irregular flake and granular structures, respectively. When the magnification was 5000×, CCSP-1 (B) presented relatively smooth granular aggregates with some filaments. The surface of CCSP-2 (D) was relatively rough, and particles of different sizes were tightly gathered together, and there were a few filaments between the particles. CCSP-3 (F) presented irregular flakes with messy filaments, and some flakes had a small hole.

### 3.3. Immunomodulatory Activities of CCSP-2 in the CTX Treated Mice

#### 3.3.1. Effect of CCSP-2 on Body Weight and Organ Index

CCSP-2 was chosen to further evaluate its in vivo immunomodulatory activity on the grounds that it had the highest yield and the strongest in vitro antioxidant capacity (data not shown).

Table 2 displays the effects of CCSP-2 on the increase of body weight, spleen index and thymus index in the CTX-induced immunosuppressed mice. Compared with the normal mice, the increase of body weight in the model group mice was strikingly decreased (*p* < 0.05), while those of the low-, medium- and high-dose CCSP-2 groups mice were significantly increased (*p* < 0.05) as compared with the normal model group. Furthermore, there was no significant difference in the increase of body weight among the medium- and high-dose CCSP-2 groups and the normal group (*p* > 0.05).

Thymus and spleen are two major immune organs in mammals, and organ index is considered to be a preliminary indicator of the immune function. The spleen index and thymus index in the model group mice were remarkably lower than those of the normal group (*p* < 0.05), indicating that CTX affected the innate immune function of mice. The spleen index and thymus index in the CCSP-2 treated mice were considerably improved by contrast with the model group mice (*p* < 0.05), dose-dependently. No significant difference in the thymus index existed between the normal group and the high-dose CCSP-2 group (*p* > 0.05). In addition, Lentinan also notably increased body weight, spleen index and thymus index (*p* < 0.05).

#### 3.3.2. Effect of CCSP-2 on White Blood Cells, Neutrophils, Lymphocytes and Platelets in Peripheral Blood

Bone marrow suppression is a common side effect during chemotherapy. The main symptom is a decrease in white blood cell counts, accompanied by a decrease in platelet counts. The white blood cell and platelet counts are measured to determine whether bone marrow suppression has occurred during and after chemotherapy. As exhibited in Table 3, CTX caused a remarkable decrease in the white blood cells, neutrophils, lymphocytes and platelets counts in the peripheral blood as compared with the normal group (*p* < 0.05), indicating that CTX caused a serious bone marrow suppression. Those of the CCSP-2 treated groups were markedly improved by contrast with the model group (*p* < 0.05). Interestingly, no significant difference was observed between the normal group and the high-dose CCSP-2 group (*p* > 0.05), suggesting that CCSP-2 alleviated the CTX-induced bone marrow suppression. Furthermore, Lentinan also remarkably dwindled the CTX-induced bone marrow suppression.

#### 3.3.3. Effect of CCSP-2 on Peritoneal Macrophage Phagocytosis

**Figure 4:**
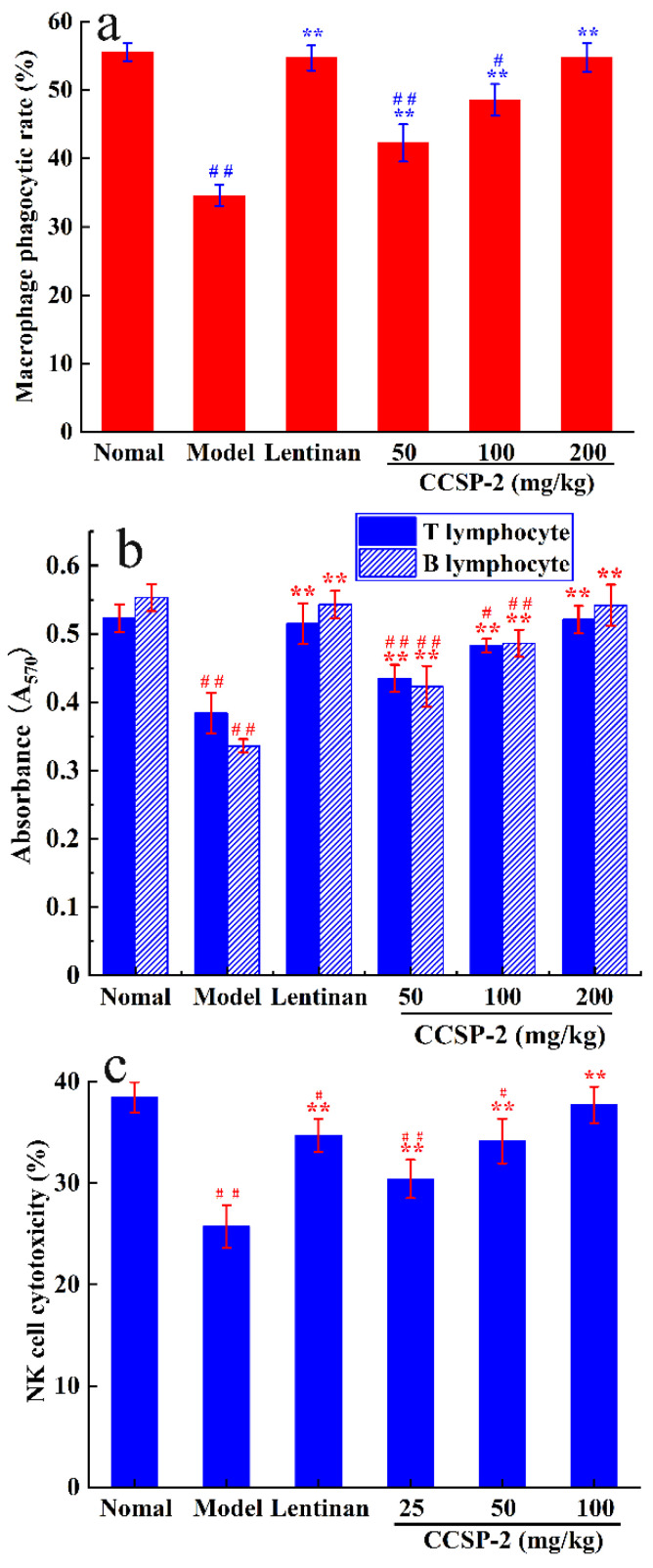
Effects of CCSP-2 on the macrophages phagocytosis (**a**), splenic lymphocyte proliferation (**b**), and NK cell cytotoxicity (**c**) in the CTX-induced immunosuppressed mice. Lentinan was employed as positive control. Results are expressed as means ± SD (*n* = 10). ** *p* < 0.01, in contrast to model group. ^#^ *p* < 0.05 and ^##^ *p* < 0.01, in contrast to normal group.

The effect of CCSP-2 on the phagocytic capacity of peritoneal macrophages in the CTX treated mice is exhibited in Figure 4a. There was a remarkable difference in macrophage phagocytic rate between model control mice and normal control mice (*p* < 0.01). The macrophage phagocytic rates in the CCSP-2 treated groups were notably higher than that of the model group (*p* < 0.01). In addition, the phagocytic rate in the high-dose CCSP-2 group was restored to normal (*p* > 0.05). Lentinan also significantly increased the macrophage phagocytosis by contrast with the model group (*p* < 0.01).

#### 3.3.4. Effect of CCSP-2 on Splenic Lymphocyte Proliferation

It is well known that Con A and LPS could induce T and B lymphocyte proliferation, respectively. The effect of CCSP-2 on splenic lymphocyte proliferation in CTX-treated mice was determined by the MTT method, as presented in Figure 4b. Compared with the normal group, CTX significantly inhibited Con A-induced T lymphocyte proliferation, as well as LPS-induced B lymphocyte proliferation (*p* < 0.01). CCSP-2 treatment strikingly increased the Con A-induced T lymphocyte proliferation and LPS-induced B lymphocyte proliferation (*p* < 0.05 or *p* < 0.01), dose-dependently. The results suggest that CCSP-2 can exert its immunoregulatory effect by promoting the T and B lymphocytes proliferation in the CTX-induced immunosuppressed mice. Lentinan also significantly promoted the splenic lymphocyte proliferation as compared with the model group (*p* < 0.01).

#### 3.3.5. Effect of CCSP-2 on NK Cell Cytotoxicity

The effect of CCSP-2 on NK cell cytotoxicity in CTX-induced immunosuppressive mice was measured by the MTT method, with splenic lymphocytes as effector cells and YAC-1 cells as target cells, as displayed in Figure 4c. After intraperitoneal injection of CTX, the NK cell cytotoxicity was notably lower than that of the normal group (*p* < 0.01). It is apparent that the NK cytotoxicity in the CCSP-2 groups was noticeably boosted (*p* < 0.01), dose-dependently. Moreover, no statistical significance was observed between the normal control and the high-dose CCSP-2 group (*p* > 0.05), hinting that CCSP-2 improved the cellular immune function in the CTX-induced immunosuppressive mice. The NK cytotoxicity was also remarkably increased in the Lentinan treated group (*p* < 0.01).

#### 3.3.6. Effect of CCSP-2 on Serum Cytokine and Immunoglobulin

**Figure 5:**
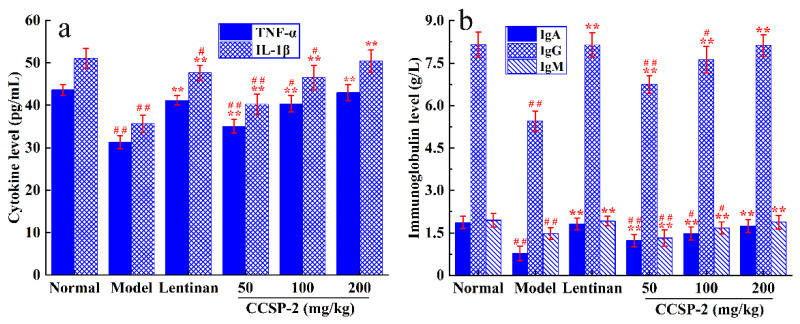
Effects of CCSP-2 on the serum cytokine (**a**) and immunoglobulin (**b**) in the CTX-induced immunosuppressed mice. Lentinan was employed as positive control. Results are expressed as means ± SD (*n* = 10). ** *p* < 0.01, in contrast to model group. ^#^ *p* < 0.05 and ^##^ *p* < 0.01, in contrast to normal group.

The effects of CCSP-2 on serum cytokines and immunoglobulins in the CTX-induced immunosuppressed mice were determined by enzyme-linked immunosorbent assay (ELISA), as displayed in Figure 5a,b. CTX significantly inhibited the serum IL-1β, TNF-α, IgA, IgG and IgM levels in mice by contrast with the normal group (*p* < 0.01). The expression levels of serum cytokines and immunoglobulins in the CCSP-2 groups mice were considerably up-regulated (*p* < 0.01), in a dose-dependent manner. No statistical difference was evident between the normal group and the high-dose CCSP-2 group (*p* > 0.05). In addition, Lentinan also remarkably up-regulated the expression levels of serum cytokines and immunoglobulins (*p* < 0.01).

#### 3.3.7. Antioxidant Activity of CCSP-2 in CTX-Induced Immunosuppressed Mice

**Figure 6:**
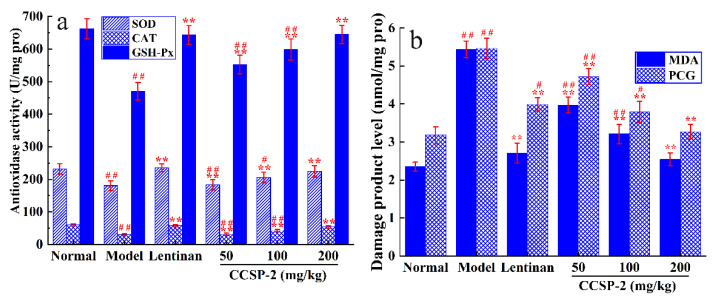
Effects of CCSP-2 on antioxidase activity (**a**) and oxidative damage product level (**b**) of liver tissue in the CTX-induced immunosuppressed mice. Lentinan was employed as positive control. Results are expressed as means ± SD (*n* = 10). ** *p* < 0.01, in contrast to model group. ^#^ *p* < 0.05 and ^##^ *p* < 0.01, in contrast to normal group.

Figure 6a shows the effect of CCSP-2 on antioxidase activity of liver tissue in the CTX-induced immunosuppressed mice. The activities of SOD, CAT and GSH-Px in the CTX group mice were remarkably lower than those of the normal group (*p* < 0.01), indicating that CTX caused damage to the antioxidant enzyme system of liver tissue. CCSP-2 treatment significantly increased the SOD, CAT and GSH-Px activities in contrast to the model control (*p* < 0.01), dose-dependently. In the high-dose CCSP-2 group, the antioxidase activities could restore to normal (*p* > 0.05), suggesting that CCSP-2 can modulate the antioxidant enzyme system of liver tissue in the CTX-induced immunosuppressed mice. Lentinan also notably increased the SOD, CAT and GSH-Px activities (*p* < 0.01).

The effect of CCSP-2 on MDA and PCG levels of liver tissue is exhibited in Figure 6b. Compared with the normal group, the MDA and PCG levels in the model group were remarkably increased (*p* < 0.01), indicating that CTX led to oxidative damage of lipids and proteins. The MDA and PCG levels in the CCSP-2-treated groups were considerably reduced (*p* < 0.01), in a dose-dependent manner, and those of the high-dose CCSP-2 group restored to normal, suggesting that CCSP-2 could alleviate the CTX-induced oxidative damage of liver tissue. The MDA and PCG levels in the Lentinan group was also notably dwindled (*p* < 0.01).

## 4. Discussion

The purification of polysaccharides is the basis for investigating their physicochemical properties, structural characterization and pharmacological function. In the present study, crude CCSP was purified by DE-52 cellulose and Sephadex G-100 chromatography affording three purified fractions, indicating that DEAE-cellulose combined with Sephadex G-100 chromatography could be applied to the purification of the crude CCSP. Although this technology is widely used in the purification of active polysaccharides, it also has some disadvantages, for instance a low flow rate, being very time-consuming and the height of the column bed occasionally changing with the ionic strength and pH value [37]. In recent years, some novel resins and gels have been developed, such as DEAE-Sepharose FF and Sephacryl. The former has the characteristics of high chemical stability and fast flow rate, and the latter has high hardness and can withstand high hydrostatic pressure. In future studies, we could attempt to employ DEAE-Sepharose FF and sephacryl chromatography for purification of the crude CCSP.

It is commonly known that pure polysaccharides are considered to be homogeneous fractions with a certain range of molecular weight distribution. The common methods to identify the homogeneity of polysaccharides are gel chromatography, ultracentrifugation and electrophoresis, among which the most widely used methods is HPGPC. In this study, the HPGPC profiles show that CCSP-1, CCSP-2 and CCSP-3 are single sharp and symmetrical peaks, indicating that they are homogeneous polysaccharide fractions. There are few reports on the molecular weight of *C. cicadae* polysaccharides. The molecular weights of two heteropolysaccharides purified by DEAE-52 and Sephadex G-100 column were determined as 3.09 × 10^4^ Da and 5.55 × 10^5^ Da, respectively [11]. Meanwhile, numerous polysaccharides have been isolated from *C. militaris*, with the molecular weight ranged from 1.2 × 10^3^ Da to 4.6 × 10^5^ Da [38]. The molecular weights of CCSP-2 and CCSP-3 were similar to those of the aforementioned polysaccharides, but that of CCSP-1 was unusually high. It is believed that the molecular weight of polysaccharides is closely bound up with their pharmacological function. An array of researchers asserts that a high molecular mass of polysaccharide is indispensable for its immunomodulatory and antitumor activities [39]. In contrast, other studies have revealed that some low molecular weight polysaccharides also have good biological activities [40,41,42]. Moreover, *Ganoderma lucidum* polysaccharide fraction with high molecular weight has better immunoenhancement effect, while fraction with low molecular weight possesses better anti-inflammatory activity [43]. In the current study, the molecular weights of CCSP-1, CCSP-2 and CCSP-3 are considerably different, and the difference in the biological activity requires further investigation.

In the present study, GC-MS was used to determine monosaccharide composition. Compared with GC method, the results of GC-MS method is more vivid by matching the acquired spectra against the mass spectral library. A large number of studies apply the same method to determine the monosaccharide composition, such as *Annona squamosa* polysaccharide [44], *Cyclocarya paliurus* polysaccharide [45], *Coriolus versicolor* polysaccharide [46] and *Lepidium meyenii* polysaccharide [47]. Many studies have shown that monosaccharide composition is closely related to biological activity [48,49,50]. It has been demonstrated that the main monosaccharide component of most active polysaccharides is glucose, followed by galactose, mannose and xylose. In the present study, glucose was found to be the predominant monosaccharide in CCSP-1, CCSP-2 and CCSP-3.

Polysaccharides are widely acknowledged as the most famous and most effective mushroom-derived biologically active substances with antitumor and immunomodulatory activities [51]. The main underlying mechanisms by which natural polysaccharides exert their antitumor activity in vivo are generally considered to be improving host immune responses [39,52]. These findings have drawn considerable attention to investigate whether CCSP-2 could be impoldered as a promising natural immunomodulator. In the current study, a CTX-induced immunosuppressive mice model was employed to observe the protective effect of CCSP-2 from the aspects of immune organ, immune cell, and immune molecule. Normal immunocompetent mice were intraperitoneally administered with 120 mg/kg CTX every day for three consecutive days, which caused immunosuppression and oxidative stress. CTX treatment significantly reduced the immune organ indices, caused bone marrow suppression, decreased macrophage phagocytic activity, splenic lymphocyte proliferation ability and NK cytotoxicity, down-regulated serum cytokine and immunoglobulin levels, and led to a decrease in antioxidase activities and an increase in oxidative damage product levels. These results seem to be in line with previous reports [53,54,55].

Macrophages, the first line of defense in the innate immune system, not only play the roles of vanguard against pathogen invasion and communication soldier for activation of innate immunity, but also participate in adaptive immunity. An increase in phagocytic capacity is regarded as a hallmark of macrophage activation [56]. Lymphocytes are regarded as an important key in cellular immunity and humoral immunity. Lymphocyte proliferation is a critical feature of lymphocyte activation, which is also considered to be a landmark event in the activation of adaptive immunity [57]. NK cells are considered to be one of the fastest cell types to arrive at inflammation sites, which can lyse tumor and pathogen-infected cells [58,59]. In this study, CCSP-2 significantly enhanced the phagocytic activity of macrophages, stimulated Con A-induced T lymphocyte proliferation and LPS-induced B lymphocyte proliferation, and improved NK cytotoxicity in CTX-induced immunosuppressive mice, in a dose-dependent manner. This observation hinted that CCSP-2 exerted its protective effect on CTX-induced immunosuppressive mice by enhancing not only innate immunity, but also adaptive immunity.

IL-1β is mainly produced by macrophages, monocytes and dendritic cells, which exerts a crucial role in the body’s immune response against infection [60]. When innate immune cells are exposed to pathogen-associated molecular patterns (PAMPs) or other alarmins that bind to toll-like receptors (TLRs) or RIG-like receptors (RLRs), the transcription factor NF-κB is activated, and the IL-1β precursor is synthesized [61]. The IL-1β precursor is required to be cleaved by caspase-1, and the latter has to be activated by inflammasomes mediated by pattern recognition receptors (PRRs) in the cytoplasm [62]. TNF-α is mainly produced by activated macrophages and is involved in various physiological processes such as immune regulation and inflammatory response [63]. It is considered to be one of the most promising anti-tumor cytokines, which exerts antitumor activity via induction of immune responses, apoptosis of tumor cells and destruction of tumor vascular-network [64]. Immunoglobulins are the main components of humoral immunity, which can activate complement, specifically bind to antigen, and play a direct role in neutralizing exotoxins, resisting viral infection and inhibiting bacterial adhesion to host cells [65]. IgA, IgG and IgM are the most abundant immunoglobulins in the body. In the present study, CCSP-2 can restore serum IL-1β, TNF-α, IgA, IgG and IgM levels, suggesting that CCSP-2 can exert its protective effect in CTX-induced immunosuppressive mice by regulating the cytokines secretion levels and enhancing the body’s humoral immunity response.

The liver is considered to be the main metabolic site of CTX in mammals. A large amount of ROS will be produced during the biotransformation of CTX, which damages the antioxidant system and leads to the decrease of antioxidant enzyme activities such as SOD, CAT and GSH-Px [66]. The antioxidant enzyme activity can reflect the degree of oxidative damage. The excessive ROS could also attack biomolecules within cells, resulting in cell death and tissue damage [67]. MDA and PCG are the sensitive biomarkers of oxidative damage to lipids and proteins [68,69]. Our results showed that CCSP-2 could increase the SOD, CAT and GSH-PX activities, and reduce the MDA and PCG levels in liver tissues, indicating that CCSP-2 could improve the antioxidant capacity of CTX-induced immunosuppressed mice.

In this study, Lentinan was employed as a positive control. The findings showed that Lentinan can activate macrophages, lymphocytes and NK cells, regulate the expression of cytokines and immunoglobulins, and improve the host’s antioxidant capacity, which are consistent with previous reports [70,71,72]. In the above immunomodulatory activity experiments, the ameliorative effect of high-dose CCSP-2 in CTX-induced immunosuppressive mice is almost equivalent to that of Lentinan, indicating that CCSP-2 could be developed as a potential immunomodulator. Studies have shown that GLSP exerts potent immunomodulatory and antioxidant effects. Guo et al. reported that GLSP can inhibit the secretion of TNF-α and IL-6 in peritoneal macrophages, potentiate the Con A-induced splenocytes proliferation [73]. Another study showed that GLSP could increase the liver and spleen levels of total antioxidant capability, glutathione reductase and CAT, and promote the serum levels of IL-2, Ig A and IgG in broilers [74]. Wang et al. found that GLSP can promote the Con A or LPS induced splenocytes proliferation, enhance NK cytotoxicity, augment phagocytic capacity of macrophages in S180-bearing mice [20]. Our results are consistent with these studies.

## 5. Conclusions

Three polysaccharide fractions CCSP-1, CCSP-2 and CCSP-3 were isolated from spores of *C. cicadae* with molecular weights of 1.79 × 10^6^, 5.74 × 10^4^ and 7.93 × 10^3^ Da, respectively. CCSP-2 consisted of mannose and glucose. CCSP-2 had the capability to improve the immune organ index, activate macrophages, stimulate T and B lymphocytes proliferation, enhance NK cytotoxicity, improve bone marrow suppression, up-regulate TNF-α and IL-1β levels, boost IgA, IgG and IgM levels, increase SOD, CAT and GSH-Px activity, and reduce MDA and PCG levels. These results reveal that CCSP-2 exerts ameliorative effects against CTX-induced toxicity in mice through its immunomodulatory and antioxidant activities. Taken together, these findings provide that CCSP-2 might be exploited as a promising natural immunomodulator for chemotherapy-induced immunosuppression and oxidative stress. However, there are still some unresolved questions in this study. What are the signaling pathways involved in macrophages and lymphocytes activation? What is the structure–activity relationship? These issues require to be addressed. In addition, there is still a lot of work to be done on the structural characterization of CCSP-2. In future studies, a combined structural characterization method of partial degradation–methylation–NMR will be attempted to finely characterize the structure of CCSP-2.

## Figures and Tables

**Table 1:** Chemical compositions and molecular weights of crude polysaccharides from spores of *C. cicadae* (CCSP), CCSP-1, CCSP-2 and CCSP-3.

Sample	Carbohydrate (%)	Protein (%)	Uronic Acid (%)	Molecular Weight (Da)	Sugar Components (%)			
					Xylose	Mannose	Glucose	Galactose
Crude CCSP	74.15 ± 3.23	1.23 ± 0.08	1.64 ± 0.13		1.21	4.03	91.96	2.80
CCSP-1	97.94 ± 4.84	- ^a^	0.16 ± 0.02	1.79 × 10^6^	-	12.29	80.46	7.25
CCSP-2	96.65 ± 5.32	-	1.15 ± 0.18	5.74 × 10^4^	-	5.73	94.27	-
CCSP-3	90.43 ± 4.60	3.18 ± 0.05	3.46 ± 0.24	7.93 × 10^3^	22.28	2.05	63.40	12.27

Results are represented as means ± SD (*n* = 3). ^a^ Not detectable.

**Table 2:** Effects of CCSP-2 on increase of body weight, spleen index and thymus index in the CTX-induced immunosuppressed mice.

Group	Dose (mg/kg)	Increase of Body Weight (g)	Spleen Index (mg/g)	Thymus Index (mg/g)
Normal	-	7.85 ± 0.47	5.26 ± 0.35	2.78 ± 0.26
Model	-	4.52 ± 0.43 ^#^	4.32 ± 0.31 ^#^	1.54 ± 0.29 ^#^
Lentinan	150	7.28 ± 0.55 *	5.15 ± 0.22 *^,#^	2.65 ± 0.13 *
CCSP-2	50	6.13 ± 0.41 *^,#^	4.87 ± 0.29 *^,#^	2.24 ± 0.12 *^,#^
CCSP-2	100	7.42 ± 0.50 *	5.18 ± 0.26 *^,#^	2.63 ± 0.15 *^,#^
CCSP-2	200	7.76 ± 0.61 *	4.22 ± 0.25 *^,#^	2.69 ± 0.21 *

Results are expressed as means ± SD (*n* = 10). Lentinan was employed as positive control. * *p* < 0.05, compared with model group. ^#^ *p* < 0.05, compared with normal group.

**Table 3:** The effect of CCSP-2 on white blood cells, neutrophils, lymphocytes and platelets counts in peripheral blood.

Group	Dose (mg/kg)	White Blood Cells (×10^9^/L)	Neutrophils (×10^9^/L)	Lymphocytes (×10^9^/L)	Platelets (×10^11^/L)
Normal	-	9.49 ± 1.24	1.25 ± 0.13	7.48 ± 0.92	6.64 ± 1.15
Model	-	4.86 ± 0.57 ^#^	0.67 ± 0.09 ^#^	3.55 ± 0.79 ^#^	3.36 ± 0.83 ^#^
Lentinan	150	7.35 ± 0.93 *^,#^	1.16 ± 0.11 *	7.02 ± 0.64 *	6.62 ± 0.95 *
CCSP-2	50	6.55 ± 0.84 *^,#^	0.77 ± 0.08 *^,#^	4.67 ± 0.86 *^,#^	4.47 ± 0.73 *^,#^
CCSP-2	100	7.98 ± 1.02 *^,#^	0.96 ± 0.09 *^,#^	6.53 ± 1.24 *^,#^	5.61 ± 1.04 *
CCSP-2	200	8.96 ± 1.15 *	1.14 ± 0.17 *	7.28 ± 1.23 *	6.63 ± 1.25 *

Results are expressed as means ± SD (*n* = 10). Lentinan was employed as positive control. * *p* < 0.05, compared with model group. ^#^ *p* < 0.05, compared with normal group.

## Data Availability

Data is contained within the article.
